# The Structure of Microbial Community and Degradation of Diatoms in the Deep Near-Bottom Layer of Lake Baikal

**DOI:** 10.1371/journal.pone.0059977

**Published:** 2013-04-01

**Authors:** Yulia R. Zakharova, Yuri P. Galachyants, Maria I. Kurilkina, Alexander V. Likhoshvay, Darya P. Petrova, Sergey M. Shishlyannikov, Nikolai V. Ravin, Andrey V. Mardanov, Alexey V. Beletsky, Yelena V. Likhoshway

**Affiliations:** 1 Limnological Institute, Siberian Branch, Russian Academy of Sciences, Irkutsk, Russia; 2 Bioengineering Center, Russian Academy of Sciences, Moscow, Russia; Missouri University of Science and Technology, United States of America

## Abstract

Insight into the role of bacteria in degradation of diatoms is important for understanding the factors and components of silica turnover in aquatic ecosystems. Using microscopic methods, it has been shown that the degree of diatom preservation and the numbers of diatom-associated bacteria in the surface layer of bottom sediments decrease with depth; in the near-bottom water layer, the majority of bacteria are associated with diatom cells, being located either on the cell surface or within the cell. The structure of microbial community in the near-bottom water layer has been characterized by pyrosequencing of the 16S rRNA gene, which has revealed 149 208 unique sequences. According to the results of metagenomic analysis, the community is dominated by representatives of Proteobacteria (41.9%), Actinobacteria (16%); then follow Acidobacteria (6.9%), Cyanobacteria (5%), Bacteroidetes (4.7%), Firmicutes (2.8%), Nitrospira (1.6%), and Verrucomicrobia (1%); other phylotypes account for less than 1% each. For 18.7% of the sequences, taxonomic identification has been possible only to the Bacteria domain level. Many bacteria identified to the genus level have close relatives occurring in other aquatic ecosystems and soils. The metagenome of the bacterial community from the near-bottom water layer also contains 16S rRNA gene sequences found in previously isolated bacterial strains possessing hydrolytic enzyme activity. These data show that potential degraders of diatoms occur among the vast variety of microorganisms in the near-bottom water of Lake Baikal.

## Introduction

Studies on the diversity of the microbial community colonizing diatoms can provide an insight into the role of microorganisms in degradation of diatoms and turnover of biogenic elements, including Si. In the world ocean, bacteria have been shown to colonize fresh diatom detritus [1], living diatoms [2], and sea snow aggregates [3]. They utilize approximately half of organic matter from primary production [4] with the aid of hydrolytic enzymes [2], [3]. Secreted extracellular polysaccharides, organic components of the cell wall, and cell contents can serve as substrates for the development of heterotrophic bacteria in algal–bacterial communities.

In the pelagic zone of the world ocean, approximately 40% of photosynthetically fixed carbon is expended for producing extracellular polymeric substances (EPS) [5]–[7]. Polysaccharides are the main constituent of EPS [8]–[13], which also contain several percent of proteins [9], [14]. In freshwater ecosystems, protein and polysaccharide components of diatom EPS can be utilized for bacterial growth, which has been confirmed in model experiments [15], [16].

The organic casing of diatom cell walls [17] is decomposed by proteases produced by colonizing bacteria, which leads to accelerated dissolution of siliceous diatom frustules [18]. In marine diatoms, the *in situ* dissolution rate of diatom silica has been shown to increase significantly under the effect of natural marine bacteria representing specific phylotypes of α-, ß-, γ-Proteobacteria, the Cytophaga–Flavobacterium–Bacteroides (CFB) group, Actinobacteria, and Firmicutes [18]–[20].

Lake Baikal, situated in a center of Eurasia, is one of the world’s more unusual freshwater ecosystems. Estimated to be over 25 million years old, Lake Baikal is also the world’s deepest (maximum depth 1642 m) and largest lake, in terms of water volume (23 015 km^3^), containing some 20% of the world’s surface freshwater. Fossil diatom remains from the bottom sediments of Lake Baikal are widely used for paleoclimatic and paleolimnological reconstructions, and deeper insight into the factors responsible for their preservation or destruction may significantly contribute to the accuracy of the results. As shown by scanning electron microscopy, diatom frustules from Baikal bottom sediments are preserved to different degrees [21]. Moreover, some diatom species fail to descend to the lake bottom [22]–[24]. For example, *Synedra acus* is a member of the dominant assemblage of recent Baikal phytoplankton, but its remains in the upper layer of bottom sediments can be found not in all regions of the lake [24]–[26], because this diatom is subject to considerable degradation both in the water column [27] and in the surface sediment layer [23]. On the other hand, diatom records from Postglacial [28], [29] and Pleistocene sediments [30]–[33] are characterized by “*Synedra* peaks”, or aggregations of siliceous frustules of this diatom, which are used for biostratigraphic correlation of core samples [28].

Diatoms that long remain in the near-bottom layer before being buried in bottom sediments can serve as a substrate for various microorganisms [34]. Several bacterial strains that we recently isolated from the deep near-bottom water of Lake Baikal were found to possess hydrolytic enzyme activities and suppress the growth of *S. acus* culture [35]. However, culturing methods alone are obviously insufficient for comprehensive characterization of microbial communities associated with diatoms. Their structure can be studied by analyzing clone libraries of 16S rRNA genes [16], [36]–[38], but the scope of this method is as yet limited to several tens to hundreds of 16S rRNA gene sequences.

Metagenomic analysis [39] has been used to evaluate the diversity of microorganisms in marine ecosystems [40]–[44], thermal springs [45], [46], and fresh water bodies [47], including sites with gas hydrate-bearing sediments in Lake Baikal [48].

The purpose of this study was to characterize microbial diversity in Baikal near-bottom waters by means of large-scale pyrosequencing of 16S rRNA gene fragments. The results provided evidence for the presence of potential degraders of diatoms among the vast variety of microorganisms inhabiting this water layer.

## Materials and Methods

### Sampling Sites and Procedure

No specific permits were required for the described field studies. The location is not privately-owned or protected in any way. The field studies did not involve endangered or protected species.

Samples were collected with a benthic gravity corer (BGC). One core was sampled from the upper sediment layer in Southern Baikal in June 2008 (51°46′40′′ N, 104°54′33′′ E; depth 1460 m). The cores were cross-cut into 1-cm fragments, fixed with 70% ethyl alcohol, and stored in Eppendorf tubes at 4°C. For analysis of near-bottom water six cores were sampled at one site in Middle Baikal in September 2009 (52°53′46′′ N, 107°31′53′′ E, depth 1570 m). It was a year of the *Synedra acus* dominance in the spring phytoplankton of the lake. Aliquots from the near-bottom water samples (about 5 cm above the sediment surface) were taken from BGCs and pooled in one sample (total volume 2 L). For metagenomic analysis, suspended matter was collected from water samples by filtering through nitrocellulose membrane with a pore size of 0.2 µm (Sartorius, Germany), washed from the filter with 5 mL of TE buffer (10 mM Tris-HCl, 1 mM EDTA; pH 7.5) into a sterile vial, and stored at –20°C. To take microbial counts, the samples were fixed with 1% glutaraldehyde solution (Sigma, United States).

### Counts of Microorganisms in Sediments and Near-bottom Water

Cell fixation and staining followed the procedure described by Pernthaler *et al.* [49]. Briefly, a suspension of bottom sediments in sterile water (1∶ 100) was filtered through a polycarbonate membrane with a pore size of 0.2 µm (Millipore, Ireland), which was then dried, coated with 0.08% agarose, and cut into sectors. These sectors were placed on glass slides and stained with 1 ml of 1 µg/mL 4,6-diamidino-2-phenylindole (DAPI) solution in PBS : glycerol mixture (3∶ 7). Aliquots of near-bottom water samples (1 mL) were fixed with 1% glutaraldehyde solution, stained with 1 µg/mL DAPI for 2–3 min, and filtered through polycarbonate membrane (0.2 µm) using Sartorius filter units. The filters were washed with sterile water, dried in air, placed on glass slides in a drop of nonfluorescent immersion oil (MiniMed, Russia), and examined under an Axiovert 200 inverted microscope (Carl Zeiss, Germany) at an excitation wavelength of 365 nm (Osram HBO 50W/AC mercury lamp). Counts were taken in no less than 30 microscopic fields per sample; subsequent calculations were performed as described [50]. Microscopic images were made using a Penguin 600CL digital camera (Pixera Corp., United States) with the AxioSet program. The results were processed statistically [51] with the Microsoft Excel 2007.

### Scanning Electron Microscopy (SEM)

Samples of near-bottom water and sediments were centrifuged in Eppendorf tubes at 12 000 rpm for 15 min, treated with 30% hydrogen peroxide in a thermostat at 75°C for 3 h, and incubated there overnight after the thermostat was switched off. The material was then washed with distilled water, pelleted again, pipetted onto a stub for SEM, dehydrated, and sputter-coated with gold in an SDC 004 vacuum evaporator (Balzers, Liechtenstein). Preparations were examined under scanning electron microscopes Philips 525 M (Netherlands) and FEI Quanta 200 (United States).

### DNA Isolation

Total DNA was isolated as described [52], with certain modifications. Briefly, the cells were washed in TE buffer (pH 8.0), and lyzed by treating with lysozyme (1 µg/mL in 400 µL of TE buffer) at 37°C for 1 h. The lysate was then supplemented with SDS to a final concentration of 1%, incubated at room temperature for 10 min, and frozen at –20°C. After thawing at +56°C, proteins and polysaccharides were extracted with a phenol : chloroform : isoamyl alcohol mixture (25∶ 24∶ 1). Nucleic acids were precipitated from the aqueous phase by adding 0.1 volume of 3 M sodium acetate (pH 5.5) and 2.5 volumes of absolute ethanol. The mixture was incubated overnight at –20°C and centrifuged at 16 100 rpm for 30 min. The pellet was washed with two portions of 70% ethyl alcohol and dissolved in TE buffer.

### Pyrosequencing

Pyrosequencing was performed using a library of amplicons generated by PCR with universal 16S rRNA gene primers U341F (CCTACGGGRSGCAGCAG) and U515R (TTACCGCGGCKGCTGVCAC). Amplicons for pyrosequencing were made in 4 replicates and pooled in one sample. The amplicons were sequenced with a GS FLX 454 genome sequencer (Roche, USA) using Titanium reagents accoding to recommendation of the manufacturer.

### Analysis of Pyrosequencing Data

Analysis of pyrosequencing data was performed using the Mothur 1.19.0 program package [53]. The obtained sequences (Table 1, stage 1) were processed by the PyroNoise algorithm [54] to remove sequencing errors (Table 1, stage 2), and then sequences longer than 100 bp with homopolymer tracts of no more than 6 bp were selected (Table 1, stage 3) and aligned with the bacterial 16S rRNA gene sequences from the SILVA database [http://www.mothur.org/wiki/Silva_reference_files]. The NAST algorithm [55] with a k-mer length of 8 bp was employed for sequence alignment. Sequences shorter than 130 bp that did not map to the V3 region of the 16S rRNA gene (positions 6428–11 892 relative to the initial SILVA alignment) were excluded from further analysis (Table 1, stage 3). Nucleotide sequences of the 16S rRNA were deposited in NCBI Short Read Archive (SRAid: SRR653441). During the preclustering stage, sequences differing by one nucleotide were combined into clusters (Table 1, stage 4). Chimeric sequences were detected by the UCHIME algorithm [56] with standard parameters (Table 1, stage 5). In calculating the genetic distance matrix, multiple insertions or deletions represented by consecutive gaps were assumed to be the result of a single mutation event. Sequence clustering was based on UPGMA analysis of genetic distances. After clustering, the operational taxonomic units (OTUs) containing only one sequence upon clustering at a genetic distance level of 0.01 (singleton OTU_0.01_) were discarded (Table 1, stage 6).

**Table 1:** Pre-processing of pyrosequencing data.

No	Step	Total number of reads	Number of unique reads	Average readlength (bp)	Total datasize(bp×10^6^)
1	Raw data	373377	NA	190	67
2	Initial filtering, Pyronoise	166588	40731	148	24
3	Alignment of reads	157131	36569	148	23
4	Pre-clustering	157131	24433	148	23
5	Remove the chimeric sequences and contaminants	154248	23057	148	23
6	Remove the singleton OTUs	149208	18017	148	23

### Taxonomic Analysis

Taxonomic analysis was based on the Bayesian approach [57] and the taxonomy from the Ribosomal Database Project [58]. Sequence reads were clustered into OTUs with a distance cutoff of 0.03 (OTU_0.03_), and the clusters with a bootstrap support of at least 80% (after 1000 iterations) were assigned into taxa using the SILVA database (see above). The results were converted into the Newick format using the custom Perl script and visualized with the Tree Graph program [59]. The dendrogram showed only those branches that extended from nodes accounting for no less than 1% of all reads. If the number of reads classified into a terminal node was smaller, the branch was reduced to the intermediate node satisfying this criterion.

### Population Analysis

For population analysis**,** rarefaction curves were plotted by means of read sampling (with 10 000 iterations, at an interval of 1000 reads) and calculation of OTUs observed at distances of 0.01, 0.03, 0.05, and 0.07. The results were used to characterize the molecular genetic diversity of the community by calculating parameters of species richness (S), Good’s coverage, the ACE and Chao1 estimators, and Simpson’s inverse index for each of the above distances. Chao1 and ACE indices are one of the most widely used non-parametric estimators of species richness. Theory behind these indices is easily accessible [60]. Alignment with known 16S rRNA gene sequences was performed with the BLASTN program [61].

## Results

### Association of Bacteria with Diatoms and the Degree of Diatom Preservation

**Figure 1:**
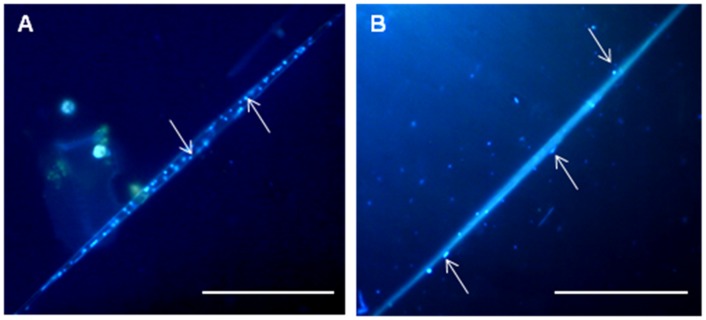
Microorganisms associated with the diatom *Synedra acus* in the near-bottom water layer of Lake Baikal. (indicated by arrows). Epifluorescent microscopy, DAPI staining. Scale bar 50 µm.

**Figure 2:**
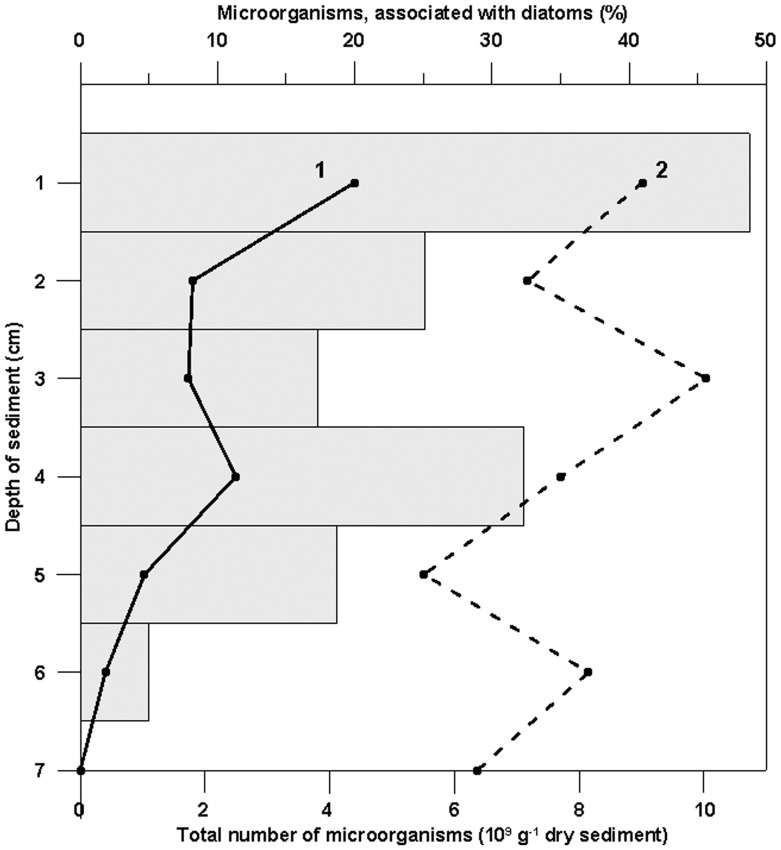
Vertical distribution of microorganisms in bottom sediments of Lake Baikal. (1) Number of diatom-associated bacteria, (2) total number of microorganisms.

**Figure 3:**
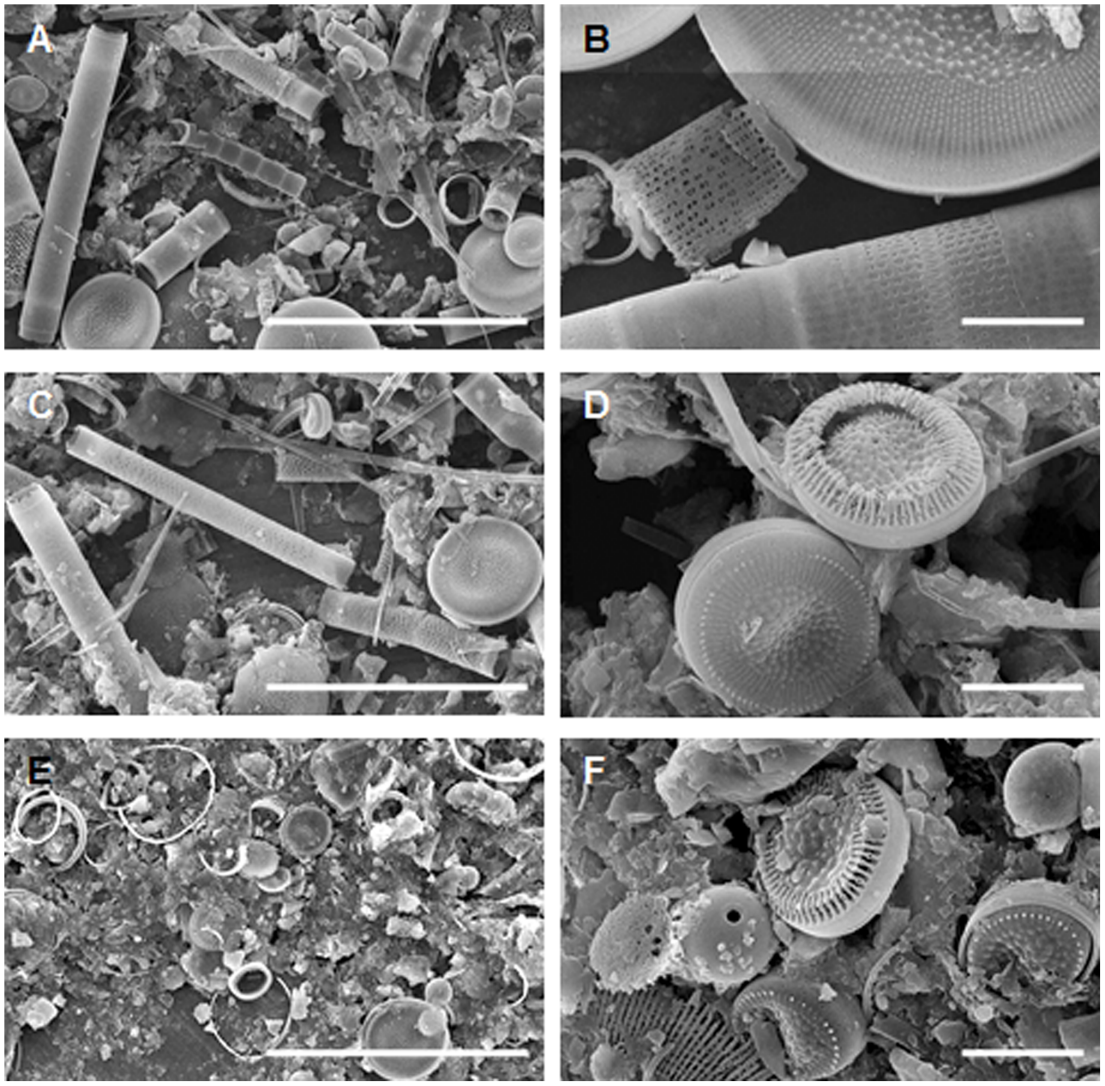
Changes in the degree of preservation of diatom frustules in different layers of Lake Baikal bottom sediments. (A, B) 0–1 cm. (C, D) 2–3 cm. (E, F) 6–7 cm. Scale bars: (A, C, E) 100 µm; (B, D, F) 10 µm.

As shown by microscopic analysis, the near-bottom microbial community was dominated by diatoms *S. acus* subsp. *radians* and free-living or diatom-associated bacteria. Figures 1A and 1B show organisms (DAPI staining – blue) which colonize the diatom *S. acus*., At the same time, chloroplasts which have to be in red under ultraviolet, are not seen. Therefore, we observed the degraded cells of diatoms and bacteria colonizing them. The latter were located either on the cell surface or within the cell (Figures 1A, B) and accounted for about 48% (6.5×10^5^ cells/mL) of the total amount of bacteria (1.4×10^6^ cells/mL). In sediment samples, diatom-associated microorganisms were distributed unevenly, with their proportion decreasing from 49% at a depth of 1 cm to zero at 7 cm (Figure 2). The degree of diatom preservation also changed with depth: frustules found in the surface layer were mainly intact (Figures 3A, B), while deeper layers (2–7 cm) contained an increasing proportion of broken and degraded frustules (Figures 3C, D, E, F).

### Metagenomic Analysis of Near-bottom Bacterial Community

Pyrosequencing of the 16S rRNA gene amplicon library resulted in more than 370 000 sequence reads with a total length of about 67×10^6^ bp (Table 1). These reads were preprocessed in order to obtain a high-quality sequence alignment providing for the minimum possible distortion in characterization of the bacterial community. As a result, the total amount of data and the number of reads were reduced by factors of about 3 and 2.4, respectively (Table 1).

**Figure 4:**
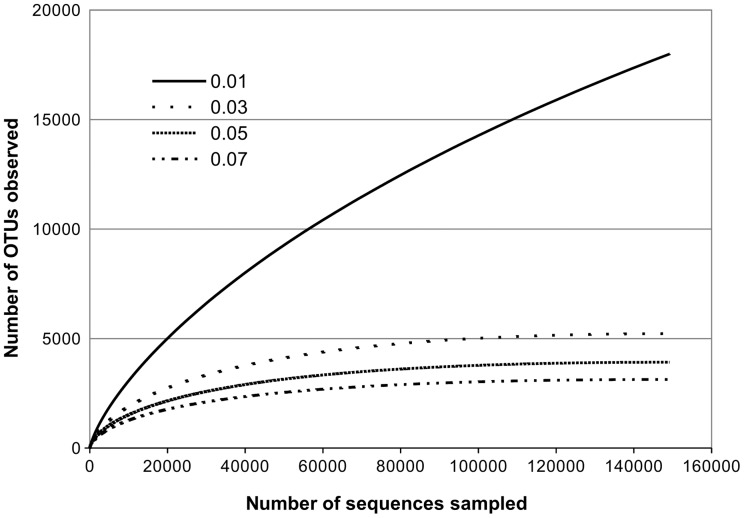
Bacterial diversity in the near-bottom water layer of Lake Baikal, as characterized by rarefaction curves of OTUs defined at genetic distance levels of 0.01, 0.03, 0.05, and 0.07.

Metagenomic analysis provided evidence for - significant molecular genetic diversity of the bacterial community from the near-bottom Baikal waters. Rarefaction curves Figure 4 and nonparametric estimators ACE and Chao (Table 2) confirmed that the sample size was sufficient for revealing 93% of OTU_0.01_ and 99.99% of OTU_0.05_ ((this clustering distance empirically corresponds to the family rank). At the same time, the abundance of certain bacterial groups accounted for a decreased species evenness in the community: the values of Simpson’s inverse index were one to two orders of magnitude lower than the observed number of OTUs at all distance levels (Table 2), indicating that the community included a number of rare bacterial species. The community richness inferred by Chao1 and ACE estimators tends to the observed number of OTUs when the clustering distance runs above 0.03. This fact suggests that the community richness observed at clustering distances above 0.03 is likely to be close to the actual numbers. Significant difference between the actual number of OTU_0.01_ and richness estimated by Chao1_0.01_ and ACE_0.01_ is attributed to the large number of minor clusters (singletons, doubletons etc.) at this distance. This, in its turn, could be the result of both pyrosequencing errors and deep sequencing strategy used.

**Table 2:** Sample coverage, species richness and species diversity indices.

Genetic distance for OTU clustering	Good’s coverage	Number of OTUs	ACE	Chao1	Simpson’s Inverse Index
0.01	0.93216	18 017	50 730 (49 846÷51 638)	34 606 (33 723÷35 539)	125.4 (123.0÷127.8)
0.03	0.99985	5 224	5 231 (5 227÷5 240)	5 224 (5 224÷5 226)	67.9 (66.7÷69.0)
0.05	0.99999	3 920	3 920 (3 920÷3 925)	3 920 (3 920÷3 920)	57.3 (56.5÷58.2)
0.07	0.99999	3 132	3 132 (3 132÷3 132)	3 132 (3 132÷3 132)	47.0 (46.4÷47.7)

### Composition of Bacterial Community

**Figure 5:**
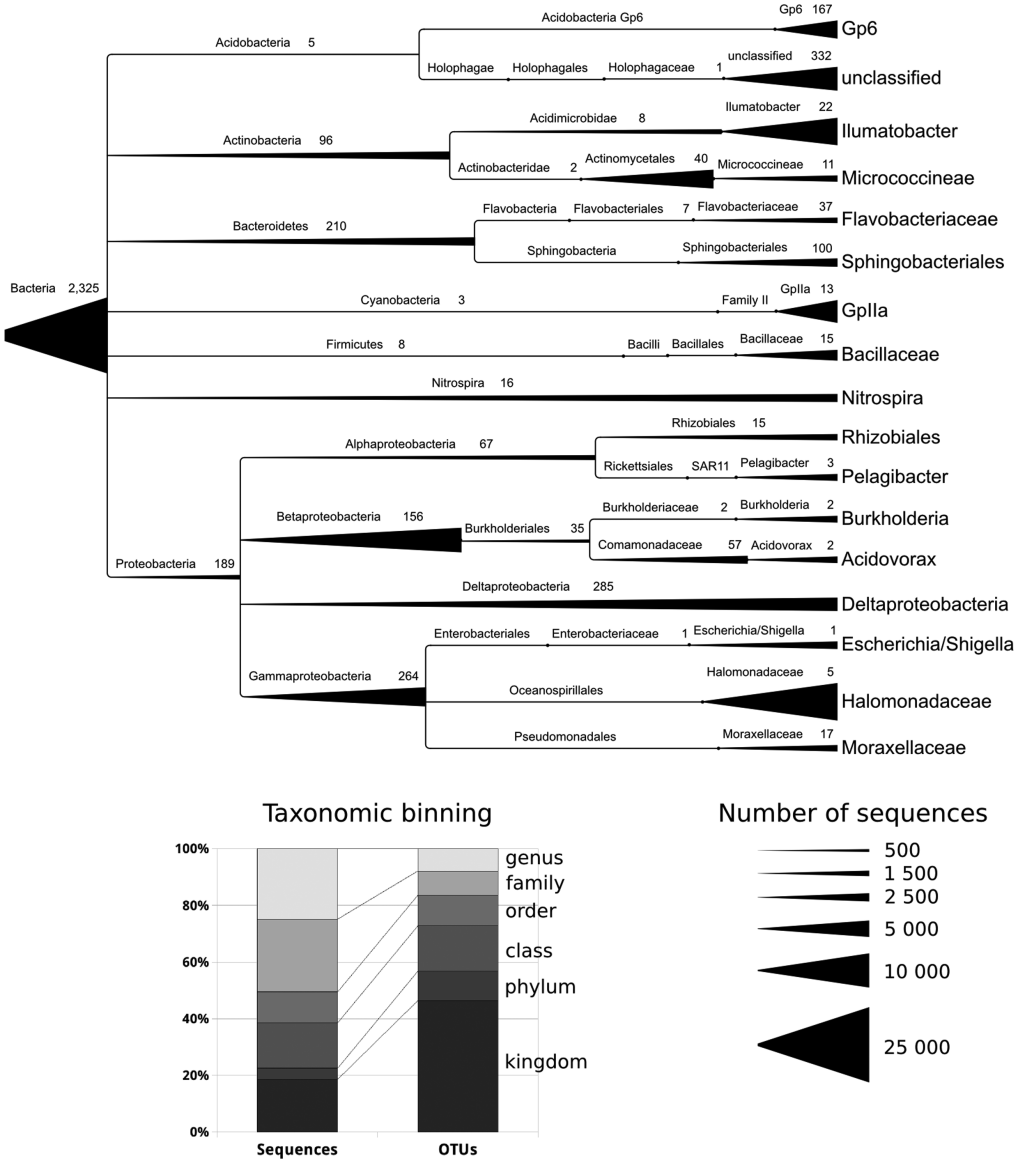
The structure of bacterial community in the near-bottom water layer of Lake Baikal. Every taxonomic group presented in the dendrogram accounted for no less than 1% of the total number of sequence reads, with the width of branches being proportional to the number of identified reads. Values at the nodes show the number of OTU_0.03_ for a given taxon. The diagram at the bottom shows the proportions of OTU_0.03_ assigned to taxa of different ranks.

As a result of taxonomic classification, approximately one-fourth of sequence reads comprising OTU_0.03_ were identified to the genus level (Figure 5). Taxonomic identification of 18% of reads and 46% of OTU_0.03_ (N = 27 881; S_0.03_ = 2426) was possible only to the Bacteria domain. Selective tests of representative sequences from the OTUs that could only be assigned to high taxonomic ranks showed that they had BLAST homologies with 16S rRNA gene fragments sequenced in DNA samples from other bacterial communities. However, the reference database provided no information on the taxonomic position of these sequences, and more detailed identification of such reads was impossible.

As follows from the results shown in Figure 5, the bacterial community in the near-bottom water layer was dominated by the members of the phylum Proteobacteria, which accounted for 41.9% of all recorded 16S rRNA gene sequences. The class α-Proteobacteria (5.7%) was represented mainly by bacteria of the genus *Pelagibacter* (about 1.3%) from the family SAR11 of the order Rickettsiales; of the genus *Caulobacter* from the family Caulobacteraceae (about 0.6%); of the order Sphingomonadales (0.9%), in which most sequences could not be identified to the genus level and only 0.2% were assigned to the genera *Sphingobium* and *Sphingomonas*; of the genus *Methylobacterium* (about 0.3%) from the family Methylobacteriaceae; and of the family Rhodobiaceae (about 0.4%). In addition, we identified microorganisms of the genus *Rhizobium* (0.2%) from the family Rhizobiaceae and of the genus *Rhodobacter* (0.2%) from the family Rhodobacteraceae. In the class β-Proteobacteria (about 14%), most sequences were classified with the order Burkholderiales (6.8%), including the genera *Burkholderia* (1.3%), *Acidovorax* (1.3%), *Methylibium* (0.2%), *Thiobacter* (0.6%), *Variovorax* (0.2%), and *Polynucleobacter* (0.2%); much smaller proportions of sequences were assigned to the orders Methylophilales (0.9%), Rhodocyclales (0.1%), and Neisseriales (less than 0.1%). In the class γ-Proteobacteria (17.2%), the dominant group included bacteria of the genus *Halomonas* from the family Halomonadaceae (8.3%); of the genera *Escherichia/Shigella*, *Serratia* from the family Enterobacteriaceae (1.6%); of the genera *Acinetobacter* and *Enhydrobacter* of the family Moraxellaceae (1.2%); of the genus *Methylobacter* from the family Methylococcaceae (0.6%); and of the genus *Pseudomonas* from the family Pseudomonadaceae (about 0.4%). Approximately 3% of bacterial sequences were assigned to δ-Proteobacteria, including the orders Bdellovibrionales (about 1%), Myxococcales (about 1%), Syntrophobacterales (0.2%), and Desulfobacterales (less than 0.1%).

The proportion of microorganisms from the phylum Actinobacteria reached 16%. They included bacteria of the genus *Ilumatobacter* from the family Acidimicrobidae (6%) and representatives of the orders Actinomycetales (6.3%) and Solirubrobacterales (about 0.9%) that were not identified in more detail.

The phylum Acidobacteria (6.9%) was represented by members of the orders Gp6 (4%), Gp4 (0.9%), Gp22 (0.7%), and Gp7 (0.4%) that were not identified to the genus level.

Representatives of the phylum Cyanobacteria accounted for about 5% of the community, almost all of them being assigned to the family GpIIa (5%).

In the phylum Firmicutes (2.8%), about 2.3% of sequences were assigned to the family Bacillaceae (including the genera *Caldalkalibacillus*, *Bacillus*, and *Oceanobacillus*) and about 0.3%, to the family Staphylococcaceae (*Staphylococcus*).

Microorganisms of the phylum Bacteroidetes comprised about 4.7% of the microbial community. These were mainly representatives of the order Flavobacteriales (1.2%), in which the genus *Flavobacterium* dominated, and of the order Sphingobacteriales (about 1.8%), including the families Chitinophagaceae, Cytophagaceae, and Sphingobacteriaceae.

Identified members of the phylum Nitrospira mostly belonged to the genus *Nitrospira* (about 1.6%). Approximately 1% of microorganisms were classified with the phylum Verrucomicrobia and assigned to the genera *Luteolibacter*, *Prosthecobacter*, and *Haloferula* from the family Verrucomicrobiaceae and the genus *Opitutus* from the family Opitutaceae. Less than 1% of sequence reads were assigned to each of the phyla Chloroflexi, TM7, and WS3, and less than 0.1%, to each of 14 other phylotypes: Planctomycetes, Chlamydiae, Deinococcus-Thermus, Gemmatimonadetes, OD1, Spirochaetes, Fusobacteria, SR1, Caldiserica, Aquificae, Thermotogae, Deferribacteres, Tenerricutes, Lentiphaerae.

In our previous experiments [35], several bacterial strains possessing different hydrolytic activities were isolated from the near-bottom water layer by culturing on the medium containing diatom cell hydrolysate as the only source of organic matter. To estimate the degree to which these bacteria are represented in the metagenome of the bacterial community, we added their full-length 16S rRNA gene sequences into alignment of the pyrocequensing reads mapped to V3 hypervariable region. The relative abundance of isolate-specific pylotypes was calculated by dividing the corresponding phylotype richness to the number of sequences in the dominated phylotype belonging to Actinobacteria (Table 3). The results showed that clusters comprising gene sequences of *Sphingomonas rhizogenes* (N_0.03_ = 472) –10.6%, *Acinetobacter johnsonii* (N_0.03_ = 425) − 9.6%, *Brevundimonas bullata* (N_0.03_ = 412) − 9.3%, *Methylobacterium adhaesivum* (N_0.03_ = 401) –9%, *Agrobacterium tumefaciens* (N_0.03_ = 171) − 3.9% were represented in the metagenome at a medium level, while clusters of *Bacillus simplex (*N_0.03_ = 10) − 0.2% and *Deinococcus aquaticus* (N _0.03_ = 5) − 0.1% were minor.

**Table 3:** The bacteria from the GenBank database most closely related, according to 16S rRNA gene sequences identified from deep near-bottom layer of Lake Baikal.

Phylotype	Number of sequences	Closest relative	Accession no	% Similarity	Location, setting
α-Proteobacteria	171	*Rhizobium* sp. *Agrobacterium tumefaciens*	FR870233 JF700414	100 100	Different plant speciesRhizosphere of tomato plant
	401	*Methylobacterium* sp.*Methylobacterium podarium*	JF905617 HQ220089	100 100	Soil of Barrientos Island Citrus roots in Florida
	412	*Caulobacter* sp. *Brevundimonas diminuta*	JF905609 HQ857771	100 100	Soil of Barrientos Island Bacterial soil communities
	472	*Sphingomonas* sp.	HM447771	100	Agricultural soil
	762	Uncultured α-Proteobacteria Uncultured SAR11 α-Proteobacterium	HQ532193 HM856580	100 100	Epilimnion from Brandy Lake Yellowstone Lake, USA
	67	Uncultured Rhodobacteraceae bacterium	EU642175	99	Lake Michigan
β-Proteobacteria	112	Uncultured *Methylibium* sp.	EU512961	97	Creosote contaminated soil
	138	Uncultured *Burkholderiaceae* bacterium	AM936595	96	Hydrocarbon-contaminated soil
	91	Uncultured *Acidovorax* sp.	JF460954	100	Drinking water, USA
	169	Uncultured *Curvibacter* sp.	HQ008595	99	Argentine freshwater reservoir
γ-Proteobacteria	645	*Halomonas nitritophilus*	GU113002	100	Mud volcano soil, China
	206	*Halomonas* sp.	AY962237	100	Soda Lakes
	690	Uncultured Enterobacteriaceae bacterium *Escherichia coli*	JF703628 HQ219946	100 100	Root and rhizophere soilRhizosphere of plant
	146	*Serratia* sp.	HQ694786	100	Sudbury River sediment soil
	425 425	*Acinetobacter johnsonii Acinetobacter* sp.	JF915343 JF421722	100 100	Microbiota of freshwater salmon Fish surface mucus
	83	*Aeromonas sobria Pseudomonas putida*	HM244939 AF182028	100 99	Microbiota of freshwater salmon Sea bacterial plankton
	98	*Alcanivorax* sp	JF304812	99	Bacterial soil communities
δ-Proteobacteria	402	Uncultured *Syntrophobacterales* bacterium	AM935385	99	Hydrocarbon-contaminated soil
	86	Uncultured *Desulfovibrionales* bacterium	AM936790	95	Hydrocarbon-contaminated soil
Actinobacteria	4447	Uncultured bacteriumUncultured bacteriumUncultured bacteriumUncultured bacteriumUncultured actinobacteriumUncultured actinobacteriumUncultured *Acidimicrobineae* bacteriumUncultured *Ilumatobacter* sp.	HQ625559 HQ905270 FR696973 AB594277 DQ316383 HQ532565 HM856389 HM346318	100 100 100 100 100 100 100 100	Water of LakeLake Taihu, ChinaLake Redon, SpainLake Biwa, JapanLake Stechlin, GermanyCrystal LakeYellowstone Lake, USAYellowstone Lake, USA
Acidobacteria	143	Uncultured *Acidobacteria* bacterium	AM935828	99	Hydrocarbon-contaminated soil
	518	Uncultured *Acidobacteria* bacterium	DQ648911	98	PCB contaminated soil
	349	Uncultured *Acidobacteria* bacteriumUncultured Acidobacteria bacteriumUncultured *Acidobacteria* bacterium	GU9988880 EF664105 DQ828480	98 99 97	Superficial sediment of Lake Taihu Bacterial soil communities Agricultural soil
Cyanobacteria	3400	*Cyanobium* sp.Uncultured cyanobacteriumUncultured cyanobacteriumUncultured *Synechococcus* sp.	HQ832914 EU641645 AM411878 DQ519782	100 100 99 100	Lavadores Beach, Portugal Lake Michigan, USA Lake Blaarmeersen, Belgium Lake Superior, USA
Firmicutes	1395	Uncultured *Geobacillus* sp.	AB594275	100	Lake Biwa water in reed community
	503	*Caldalkalibacillus thermarum* *Caldalkalibacillus uzonensis* *Bacillus* sp.*Geobacillus* sp.*Bacillus smithii*	AY753654 DQ221694 AB043863 FJ4295900 FJ572204	100 98 100 94 99	Hot spring in ChinaHot spring in KamchatkaHot spring in TurkeyHot spring in TurkeyHot spring in India
Bacteroidetes	545	*Flavobacterium* sp.	FR682718	100	Soil sample East Antarctica
	45	*Flavobacterium* sp.*Flavobacterium* sp. uncultured*Bacteriodetes* bacterium	FR696351 GU932945 GQ469486	97 99 98	River water, FinlandWater High ArcticAgricultural soil communities
	84	Uncultured Bacteroidetes bacterium	EF020181	99	Rhizosphere of plant
Verrucomicrobia	84	*Verrucomicrobia* bacterium*Verrucomicrobium* sp.Uncultured bacterium*Verrucomicrobiae* bacterium	HM856577 FN668203 AY752095 EF520638	100 99 100 98	Yellowstone Lake, USALake Zurich, SwitzerlandPavin Lake, FranceAdirondack Lake, USA

According to the results of BLASTN screening, the closest relatives of the identified sequences have been revealed in other aquatic ecosystems and in the soil (Table 3), which is evidence that these bacteria may be involved in similar processes occurring in different ecosystems.

## Discussion

It has been shown that the abundance of bacteria in freshwater bottom sediments reaches a peak (1.4−8.7×10^9^ cells/g air-dry weight) in the surface layer and decreases with depth [62]. According to our data, this parameters in the surface layer of Baikal bottom sediments amounted to 10^10^ cells/g air dry weight, with the greater part of bacteria, both in the sediments and in the near-bottom water layer, being associated with diatom cells (Figures 1, 2). The results of DAPI staining show that diatoms in the near-bottom water layer are colonized by microorganisms, which affect both the organic matrix on the surface of frustules and the contents of diatom cells (Figures 1A, B). It appears from Figure 1A that bacteria degrade intracellular organic matter, which in *S. acus* subsp. *radians* consists half of polyunsaturated fatty acids [63] and contains chrysolaminarin as the main polysaccharide component [64].

As shown by Maksimenko *et al.* [65] using fluorescence *in situ* hybridization (FISH) with specific probes [66], diatom-associated bacteria from Lake Baikal waters belong to the classes of α- and β-Proteobacteria, as do bacteria associated with marine planktonic diatoms [20]. Previous experiments involving comparative analysis of 16S rRNA gene clonal libraries generated from lake bacterioplankton [67] failed to provide a complete picture of taxonomic diversity in the microbial communities but allowed the authors to reveal seven sequences with a relatively high degree of similarity (85.8–94.3%) to those of α-Proteobacteria, β-Proteobacteria, γ-Proteobacteria, and Actinobacteria in samples from deep waters of Middle Baikal.

According to the results of metagenomic analysis of microbial community from the deep near-bottom water layer, where diatoms settling from the water column concentrate, this community is characterized by very high taxonomic diversity, despite specific features of its ecological niche (great depth and permanently low water temperature). It may well be that this diversity is provided for by variation in the species composition of diatoms, since their dominant groups in the Baikal phytoplankton change every year and diatoms may arrive to the bottom while still alive (at least partly) due to vertical water exchange events.

Most of 149 208 unique sequences revealed in the metagenome of this community have been identified to the family or genus level. The results of their taxonomic classification show that the bulk of the community is composed by representatives of two phylotypes, Proteobacteria and Actinobacteria (Figure 5). The identified microorganisms are mainly organotrophs with different metabolic strategies that commonly occur together in different ecological niches. Known representatives of the dominant classes of α-, β-, and γ-Proteobacteria are aerobes or facultative anaerobes utilizing organic compounds, including proteins and polysaccharides, as a source of energy [68]. According to BLASTN data analysis, their closest relatives (95–100% similarity in 16S rRNA gene sequence) occur in freshwater and marine ecosystems, lake deposits, soils, and as symbionts of eukaryotes (Table 3). Most members of Actinobacteria, the second most abundant phylotype, are also organotrophic bacteria whose functional role generally consists in decomposing complex, poorly accessible substrates at later stages of microbial succession; in addition, they are possibly involved in the synthesis and decomposition of humic substances [69]. Phylogenetically close microorganisms of this group (100% similarity in 16S rRNA gene sequence) have been identified from other lake ecosystems (Table 3).

**Figure 6:**
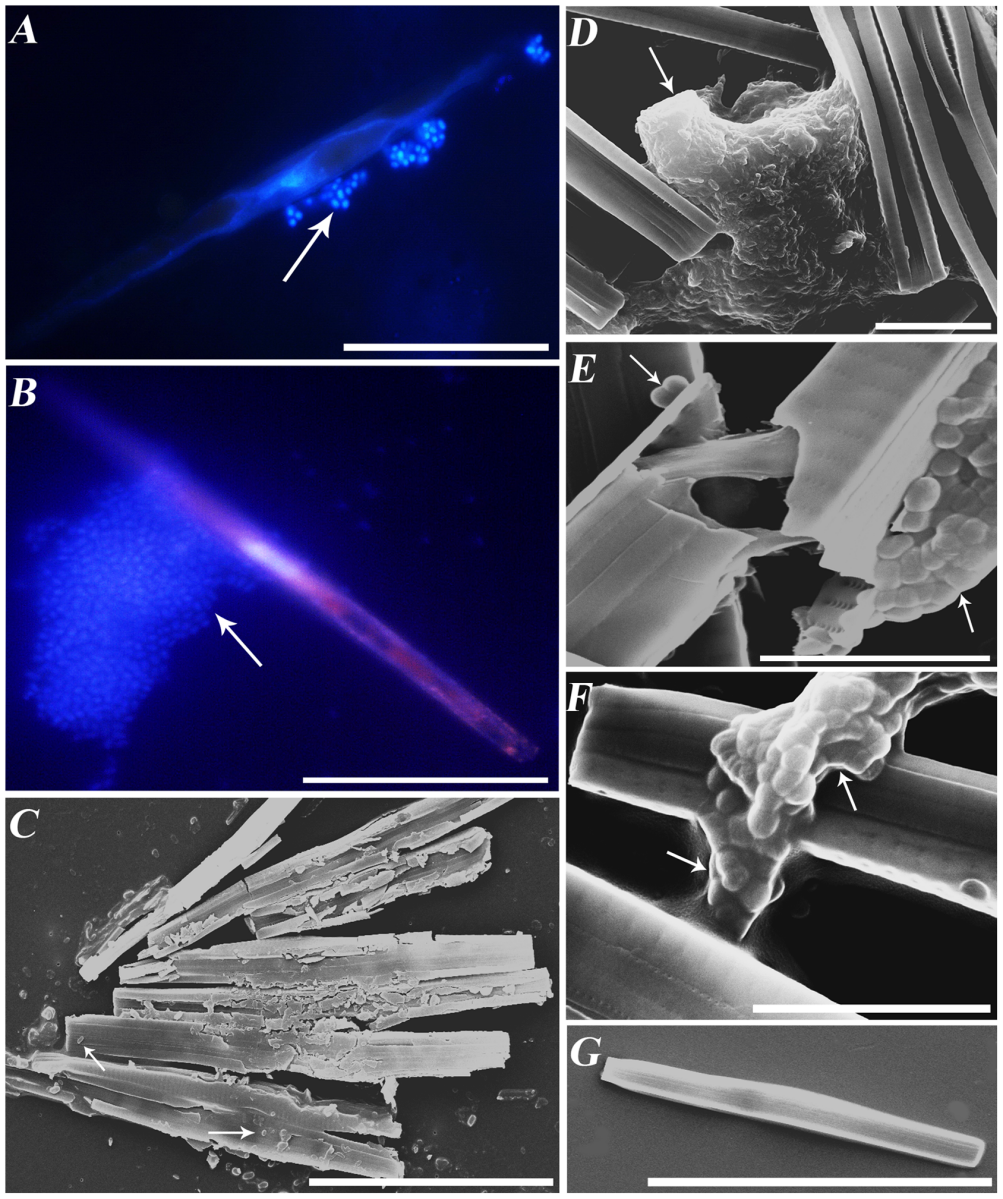
Bacterial isolates associated with the laboratory culture of *S. acus.* * A. johnsonii* BW65UT1570 (A, F), *M. adhaesivum* BW66UT1570 (B), *A. tumefaciens* BW62UT1570 (D). The degradated siliceous frustules of diatom *S. acus* in cocultures with *B. simplex* BW64UT1570 (C), *A. johnsonii* BW65UT1570 (E). Axenic culture *S. acus* (G). Epifluorescent microscopy, DAPI staining (A, B); scanning electron microscopy (D, E, F, G). Scale bar: A, B and G, 50 µm; C, 40 µm; D, 10 µm; E, F, 5 µm.

As shown in our previous study [35], isolates of *Brevundimonas bullata*, *Sphingomonas rhizogenes*, *Agrobacterium tumefaciens*, *Methylobacterium adhaesivum*, *Acinetobacter johnsonii*, *Bacillus simplex,* and *Deinococcus aquaticus* from the near-bottom Baikal water have an algicidal effect on the diatom *S. acus* and possess protease, β-xylosidase, β-glucosidase, β-galactosidase, and chitobiase activities. During joint cultivation of bacteria and the diatom S. acus we recorded inhibition of diatom growth in 4 days of incubation, whereas in 18 days siliceous frustules of *S. acus* became more brittle. Broken cells were detected in a drop under a light microscope and on a SEM slide (Figure 6). However, the degradation of diatom frustules has not been observed in the axenic culture of diatoms. Pyrosequencing reads similar to 16S rRNA gene sequences of these isolates have also been found in the metagenome of the near-bottom microbial community. Frustules of diatoms affected by bacterial hydrolytic enzymes are very brittle and easily break up even when being dried to prepare samples for SEM analysis, and the bottom sediments at a depth of 6–7 cm usually contain only macerated fragments of the frustules (Figures 3G, 3I). The involvement of bacteria in diatom degradation is indirectly confirmed by data shown in Figure 2: the proportion of bacteria associated with diatom cells in the upper layer of bottom sediments (0–7 cm) gradually decreases with depth.

However, the trend that the degree of diatom preservation decreases with depth does not apply to all Baikal diatom records. For example, Bezrukova *et al.* [70] observed that postglacial diatom sediments were strongly diluted with terrigenous material in which well-preserved diatom frustules could be found. An episode of high-rate burial of *S. acus* diatoms, which accounted for a high degree of frustule preservation, took place in the mid-Holocene [71]. Experiments on hydrogen peroxide treatment of samples from the upper sediment layer provided a basis for the conclusion that the degree of frustule preservation in bottom sediments depends on the depth of the overlying water column [21]. However, this conclusion should be revised in the light of recent data based on microscopic analysis of numerous core samples. They show that, even within the same core taken from a certain depth, the degree of diatom preservation may vary between sediment layers formed during different time periods; moreover, this parameter in the Pleistocene–Holocene cores shows no correlation with the age of sediments.

Reconstructions of hydrophysical conditions in Lake Baikal during the Late Pleistocene and Holocene [72] provide evidence for the possibility of radical changes in the hydrologic regime of the lake over the past 20 000 years, especially during glacial periods, when not only the duration of ice cover was longer and water temperature was lower, but also the pattern of vertical water mixing was probably different: the lake was monomictic rather than dimictic as it is today. Changes in the freeze-up period, variation in hydrologic conditions during the Late Pleistocene and Holocene [72], decline in phytoplankton production during glacial periods [73], increasing water turbidity and dilution of diatom flux by terrigenous runoff during the abrupt postglacial warming [69] along with possible changes in the pattern of deep-water renewal [74], [75] created different conditions for the development, settling, and burial of diatoms and, therefore, for algal-bacterial interactions. In our opinion, minimum contact with bacteria in the course of settling and burial is the main factor providing for a high degree of diatom preservation in Baikal sediments.

### Conclusion

Thus, diatoms inhabiting the deep near-bottom water layer and the upper sediment layer are colonized by bacteria that utilize the remaining organic algal material. Samples were collected from the near-bottom layer a month later after active growth of *S. acus* subsp. *radians* which occurs at certain periodicity. Metagenomic analysis revealed high taxonomic diversity of bacteria despite a peculiar characteristic of the ecological niche (large depth and constant low temperature). This diversity is likely to be attributed to the change of species composition of diatoms, the dominant complexes of which are replaced in phytoplankton of Lake Baikal every year, and the vertical water mixing makes living diatoms episodically settle to the bottom. Algicidal effect of bacterial isolates on *S. acus s*ubsp. *radians* and the presence of pyrosequencing reads similar to nucleotide sequences of these isolates attest that under natural conditions the bacterial community can degrade extracellular and intracellular organic matter of diatoms, thus accelerating processes of dissolution of biogenic silica.
